# *Toxoplasma gondii* Type-I ROP18 Targeting Human E3 Ligase TRIM21 for Immune Escape

**DOI:** 10.3389/fcell.2021.685913

**Published:** 2021-05-26

**Authors:** Lijie Yao, Liqing Xu, Lijuan Zhou, Shuizhen Wu, Weihao Zou, Min Chen, Jiating Chen, Hongjuan Peng

**Affiliations:** Guangdong Provincial Key Laboratory of Tropical Disease Research, Department of Pathogen Biology, School of Public Health, Southern Medical University, Guangzhou, China

**Keywords:** *Toxoplasma gondii*, TRIM21, *Tg*ROP18_I_, NF-κB, ubiquitin

## Abstract

*Toxoplasma gondii* is an intracellular pathogen that exerts its virulence through inhibiting host’s innate immune responses, which is mainly related to the type II interferon (IFN-γ) response. IFN-γ inducible tripartite motif 21 (TRIM21), an E3 ligase, plays an important role in anti-infection responses against the intracellular pathogens including bacteria, virus, and parasite. We found that *T. gondii* virulence factor ROP18 of the type I RH strain (*Tg*ROP18_I_) interacted with human TRIM21, and promoted the latter’s phosphorylation, which subsequently accelerated TRIM21 degradation through lysosomal pathway. Furthermore, TRIM21 protein level was found to be upregulated during RH and CEP strains of *T. gondii* infection. TRIM21 knocking down reduced the ubiquitin labeling on the parasitophorous vacuole membrane (PVM) [which led to parasitophorous vacuole (PV) acidification and death of CEP tachyzoites], and relieved the inhibition of CEP proliferation induced by IFN-γ in human foreskin fibroblast (HFF) cells which was consistent with the result of TRIM21 overexpression. On the other hand, TRIM21 overexpression enhanced the inhibition of CEP proliferation, and inhibited the binding of IκB-α with p65 to activate the IFN-γ-inducible NF-κB pathway, which might be resulted by TRIM21-IκB-α interaction. In brief, our research identified that in human cells, IFN-γ-inducible TRIM21 functioned in the innate immune responses against type III *T. gondii* infection; however, *Tg*ROP18_I_ promoted TRIM21 phosphorylation, leading to TRIM21 degradation for immune escape in type I strain infection.

## Introduction

*Toxoplasma gondii* is an obligate intracellular protozoon, and about 30% of the world population is serum positive with its antibodies (Montoya and Liesenfeld, 2004). Human infections are mostly asymptomatic, but bring a lifelong threat to the infected population because of the latent stage (bradyzoites) in the tissues and organs (Harker et al., 2015). *T. gondii* infection can cause life-threatening symptoms such as encephalitis and retinochoroiditis in immunocompromised individuals, and adverse pregnancy outcomes such as stillbirth, abortion, and tetras in pregnant women in the primary infection (Montoya and Liesenfeld, 2004; Elmore et al., 2010; Torgerson and Mastroiacovo, 2013; Hide, 2016).

During infection, *T. gondii* rhoptries discharge many effectors to the host cytoplasm and the parasitophorous vacuole (PV) to modulate the homeostasis between the host cell and the parasite (Bradley et al., 2005; Boothroyd and Dubremetz, 2008; Hakimi et al., 2017). Among these effectors, *Tg*ROP18_I_ is considered to be a key determinant related to the high mortality phenotype of type I strains (Saeij et al., 2006; Taylor et al., 2006). As a protein kinase of the ROP2 subfamily, *Tg*ROP18_I_ is discharged into the host cell and mediates some host proteins for successful parasitism (Bradley et al., 2005; Sinai, 2007). On the other hand, host cells have to trigger a rapid recognition and defense immunity to control the multiplication of the intracellular pathogens (Bianchi and van den Bogaart, 2020). The cytokine gamma interferon (IFN-γ) is induced in the early infection of the intracellular pathogens including *T. gondii* (MacMicking, 2012), which is crucial to restrain both the acute infection and chronic infection (Suzuki et al., 1988; Yap and Sher, 1999). The transcription of genes related to host immune system can be upregulated by IFN-γ treatment (Platanias, 2005); *T. gondii* can also manipulate some of these genes’ transcription in infected cells to maintain its replication (Platanias, 2005; Kim et al., 2007).

NF-κB signaling pathway, as one of the major IFN-γ-inducible signaling cascades involved in host’s innate immunity, could be regulated by *T. gondii* (Ge et al., 2014). In infected human foreskin fibroblasts (HFFs), type I *T. gondii* prohibits NF-κB activation by inhibiting p65/RelA phosphorylation and translocation to the nucleus (Shapira et al., 2005). Moreover, it has been confirmed that *T. gondii* inhibits IκB-α degradation and p65 phosphorylation, which results in an inhibition of caspase-1 cleavage in the infected neutrophils (Lima et al., 2018), but these phenomena are not found in the infected human monocytes (Gov et al., 2013, 2017). These reports imply that *T. gondii* regulates host NF-κB pathway through varied ways in different host cells.

Tripartite motif (TRIM) proteins have emerged as an important family of E3 ligases that play a versatile role in innate immunity against infection (Giraldo et al., 2020; Koepke et al., 2020; Lee et al., 2020; Wang and Hur, 2021). For instance, TRIM31 promotes the K63-linked polyubiquitination of mitochondrial antiviral signaling protein (MAVS) by inducing expression of interferons (IFNs) to against viral infection (Liu et al., 2017). TRIM 21, also known as Ro52/SS-A, is involved in innate and acquired immunity against viruses and bacteria *via* the ubiquitination of interferon regulatory factors IRF3, IRF5, IRF7, and IRF8, but little is known about its role in parasitic infection (Lazzari et al., 2014; Liu et al., 2018). Following *Salmonella typhimurium* infection, TRIM21 is also essential to promote cell death through conferring p62 ubiquitination and proteasomal degradation (Pan et al., 2016; Hos et al., 2020). Furthermore, it has been reported that TRIM21 mediates NF-κB activation to aggravate the inflammatory response in psoriasis (Yang et al., 2020).

In this research, based on the finding of the interaction of *Tg*ROP18_I_ with human TRIM21, we investigated the role of TRIM21 in *T. gondii* infection.

## Materials and Methods

### Parasite and Cell Culture

All *T. gondii* strains used in this study were maintained by serial passage of tachyzoites in HFF monolayers cultured at the condition of 37°C and 5% CO_2_. The *T. gondii* strains used in our study included Type-I strain RH, type-III strain CEP, the ROP18 knockout strain (RH-Δ*rop18*), and the recombinant CEP expressing type I ROP18 (CEP-*rop18*_I_). HFF and human embryonic kidney 293T (HEK293T) cells were grown in Dulbecco’s modified Eagle’s medium (DMEM, Invitrogen, Thermo Fisher Scientific, Shanghai, China) supplemented with 10% fetal bovine serum (FBS, Invitrogen, Thermo Fisher Scientific, Shanghai, China), and 50 mg/ml of penicillin and streptomycin.

### Detection of TRIM21 Transcription and Protein Level During RH and CEP Infection

Human foreskin fibroblast cells were cultured in 12-well plates and infected with the RH or CEP tachyzoites at Multiplication of Infection (MOI) three for 1 h or 24 h, or left uninfected. The TRIzol^TM^ Reagent (Invitrogen, Thermo Fisher Scientific, Shanghai, China) was used for total RNA extraction from HFFs (1.0 × 10^6^ cells per well), and cDNA was synthesized with Random Primer (N9) using TransScript^®^ All-in-One First-Strand cDNA Synthesis SuperMix for qPCR (One-Step gDNA Removal) (Transgen Biotech, Beijing, China) and subjected to Real-time PCR with the primers shown in Supplementary Table 1. For protein level detection, HFF cells were harvested and subjected to Western blotting.

### Identification of the Effects of TRIM21 Overexpression and Knockdown on RH and CEP Infection

Human foreskin fibroblast cells were seeded in 12-well plate and stimulated with 100 U/ml IFN-γ for 24 h, or transfected with 0.5 μg pcDNA3.1-TRIM21-HA for 24 h, or 80 nmol of si-TRIM21 (combining of si-TRIM21-1, si-TRIM21-2, and si-TRIM21-3) or si-NC for 48 h (the sequence of si-TRIM21 are shown in Supplementary Table 2), and then stimulated with 100 U/ml IFN-γ for 24 h. After these treatments, the HFF cells were infected with RH tachyzoites (MOI = 3) for 18 h, or CEP tachyzoites (MOI = 3) for 24 h. Subsequently, cell monolayers were subjected to immunofluorescence assay (IFA) to determine the average number of parasites within 100 PVs or the number of PVs containing 1, 2, 4, or 8 tachyzoites from 25 separated fields of view (He et al., 2017). Anti-SAG1 mouse monoclonal antibody (Abcam, Cambridge, United Kingdom), and Goat anti-Mouse IgG (H + L) highly cross-adsorbed secondary antibody and Alexa Fluor Plus 488 (Invitrogen, United States) were used. Experiments were repeated three times for statistical analysis.

### Verification of *Tg*ROP18_I_-TRIM21 Interaction and the Interaction Motif

HEK293T cells were seeded in T25 flasks to 90% confluence, and then transfected with pcDNA3.1-ROP18_I_-FLAG, pcDNA3.1-ROP18_I_-KD-FLAG, and pcDNA3.1-TRIM21-HA (or the truncation mutants of TRIM21) alone or in combination for 48 h, using Lipofectamine^®^ 3000 reagent (Thermo Fisher Scientific). The cell lysates were subjected to immunoprecipitation with anti-HA-Tag rabbit monoclonal antibody (Abcam, Cambridge, United Kingdom) or anti-FLAG M2 mouse monoclonal antibody (Sigma, St. Louis, MO, United States) followed by Western blotting.

### Detection of TRIM21 Phosphorylation Level in the Cells With Expression of *Tg*ROP18_I_

HEK293T cells were seeded in T25 flasks to 90% confluence, and transfected with pcDNA3.1-ROP18_I_-FLAG using Lipofectamine^®^ 3000 reagent (Thermo Fisher Scientific) for 24 h. The cell lysates were subjected to immunoprecipitation with anti-TRIM21 rabbit polyclonal antibody (Proteintech, IL, United States) followed by Western blotting.

### Identification of TRIM21 Degradation Through Lysosomal Pathway by *Tg*ROP18_I_

HEK293T cells were seeded in 6-well plate to 90% confluence, and transfected with 0, 0.6, or 1.2 μg of pcDNA3.1-ROP18_I_-FLAG for 24 h; or co-transfected with 1 μg pcDNA3.1-TRIM21-HA and the increased amounts of pcDNA3.1-ROP18_I_-FLAG (0, 0.5, or 1.0 μg) for 24 h; or co-transfected with 1 μg pcDNA3.1-TRIM21-HA and the increased amounts of pcDNA3.1-ROP18_I_-FLAG (0, 0.25, 0.5, or 1.0 μg) for 24 h. The cells were then treated with 10 μM MG132 or Leupeptin for 12 h, or left untreated. After that, the cells were harvested and the cell lysates were subjected to Western blotting for TRIM21 detection.

### Detection of the IFN-γ-Induced Ubiquitin Labeling on CEP Parasitophorous Vacuole Membrane (PVM) After TRIM21 Knockdown

Human foreskin fibroblasts were grown on coverslips in 12-well plate to 30–50% confluence. The cells were then transfected for 48 h with either 80 nmol of non-targeting small interfering RNA (si-NC) or siRNA specific for TRIM21 (si-TRIM21) (which were synthetized in RiboBio, Guangzhou, China; the sequences are shown in Supplementary Table 2), using Lipofectamine^®^ 3000 Transfection Reagent (Thermo Fisher Scientific) following the manufacturer’s instruction. The efficiency of TRIM21 knockdown was verified by Western blotting. After the following stimulation with 100 U/ml IFN-γ, the HFF cells were infected with CEP tachyzoites for 6 h, and then subjected to IFA with anti-ubiquitin FK-2 mouse monoclonal antibody (Enzo Life Sciences, NY, United States), and Goat anti-Mouse IgG (H + L) highly cross-adsorbed secondary antibody, Alexa Fluor Plus 594 (Invitrogen, United States).

### Identification of IFN-γ-Induced Ubiquitin Labeling on CEP PVM Leading to Acidification of the CEP Parasitophorous Vacuole

Human foreskin fibroblasts were grown on the coverslips in 12-well plate to 90% confluence, and stimulated with 100 U/ml IFN-γ for 24 h or not, followed by CEP infection for 2 h. HFFs were then treated with 50 mM LysoTracker^®^ Deep Red dye (Invitrogen, United States) for another 2.5 h, which specifically stained the acidic organelles in live cells including lysosomes. The infected cells were then washed with phosphate buffered saline (PBS) for three times, rinsed with ddH_2_O and mounted with mounting-oil containing DAPI. The PV labeling was observed under a fluorescence microscope (ECLIPSE Ni, NA = 1.4, Nikon, Tokyo, Japan).

### Verification of TRIM21 Binding to IκB-α and Resulting in Inhibition of p65-IκB-α Interaction

HEK293T cells were seeded in T25 flasks to 90% confluence, and then transfected with pcDNA3.1-TRIM21-HA for 24 h, or treated with 100 U/ml IFN-γ for 0, 12, or 24 h. The cells were then harvested and the cell lysates were subjected to immunoprecipitation with anti-TRIM21 rabbit polyclonal antibody (Proteintech, IL, United States) or anti-p65 mouse monoclonal antibody (Cell Signaling Technology, MA, United States). The immunoprecipitates were further analyzed by Western blotting.

### Quantitative Reverse Transcription PCR

Quantitative reverse transcription PCR (qRT-PCR) was performed with Hieff^®^ qPCR SYBR^®^ Green Master Mix (Low Rox Plus) (Yeasen, China) according to the manufacturer’s instruction. Real-time PCR was carried out on QuantStudio 6 Real-Time PCR System (Thermo Fisher Scientific, United States) using iQ SYBR Green Supermix (Bio-Rad, CA, United States), primers used were shown in Supplementary Table 1 and all primers were synthesized in GENERAY, Shanghai, China. The specificity of the PCR amplification was verified by a dissociation curve analysis. Each sample was run in triplicate and relative quantitation was determined using comparative Ct method with data normalized to the housekeeping gene, β-actin. Real-time PCR results were presented as fold change compared to the uninfected sample, and data were derived from four independent experiments.

### Western-Blot and Antibodies

The transfected or infected HEK293T, or HFF cells, were harvested and boiled in 1 × loading buffer (0.08 M Tris, pH 6.8, with 2.0% SDS, 10% glycerol, 0.1 M dithiothreitol, and 0.2% bromophenol blue). The cell lysates were loaded onto a 12% polyacrylamide gel for separation, and the proteins were then transferred to a polyvinylidene difluoride (PVDF) membrane. The membrane was blocked in blocking buffer [PBS containing 5% Bovine Serum Albumin (BSA) and 0.05% Tween-20] at room temperature for 2 h, and then incubated in the primary antibodies, followed by incubation in the secondary antibodies conjugated with horseradish peroxidase (HRP) at room temperature for 2 h, following the manufacturer’s instruction. Specific proteins on membranes were visualized by luminescence generated by using Clarity^TM^ Western ECL Substrate (Bio-Rad, CA, United States) and photographed with a ChemiDoc^TM^ Touch Imaging System (Bio-Rad, CA, United States).

The antibodies used in these experiments were anti-TRIM21 rabbit polyclonal antibody (Proteintech, IL, United States), anti-HA-Tag rabbit monoclonal antibody (Abcam, Cambridge, United Kingdom), anti-p65 rabbit monoclonal antibody (Abcam, Cambridge, United Kingdom), anti-p-p65 Ser536 rabbit monoclonal antibody (Cell Signaling Technology, Danvers, MA, United States), anti-IκB-α rabbit polyclonal antibody (GeneTex, Irvine, CA, United States), anti-ubiquitin FK-2 monoclonal antibody (Enzo Life Sciences, NY, United States), anti-ubiquitin (linkage-specific K63) rabbit monoclonal antibody (Abcam, Cambridge, United Kingdom), anti-Phospho-(Ser/Thr) rabbit monoclonal antibody (Abcam, Cambridge, United Kingdom), anti-DDDDK-Tag rabbit polyclonal antibody (Abcam, Cambridge, United Kingdom), anti-SAG1 rabbit polyclonal antibody (Abcam, Cambridge, United Kingdom), anti-GAPDH mouse monoclonal antibody (Enzo Life Sciences, NY, United States), anti-β-actin rabbit monoclonal antibody (Cell Signaling Technology, Danvers, MA, United States), and goat anti-rabbit or goat anti-mouse IgG-HRP (ABclonal Technology, Wuhan, China).

### Statistical Analysis

Statistical analyses were performed using GraphPad Prism v5 software. Differences between groups were assessed by two-tailed unpaired Student *t* test or one-way ANOVA with repeated measures when more than two groups were analyzed. The significance of *T. gondii* proliferation difference between groups was analyzed by two-way ANOVA. Differences were labeled ^∗^ (*p* < 0.05), ^∗∗^ (*p* < 0.01), ^∗∗∗^ (*p* < 0.001), and no significant difference was presented as “ns.” All experiments were repeated at least three times for statistical analysis.

## Results

### *Tg*ROP18_I_ Interacted With the PRY-SPRY Domain of TRIM21

**FIGURE 1:**
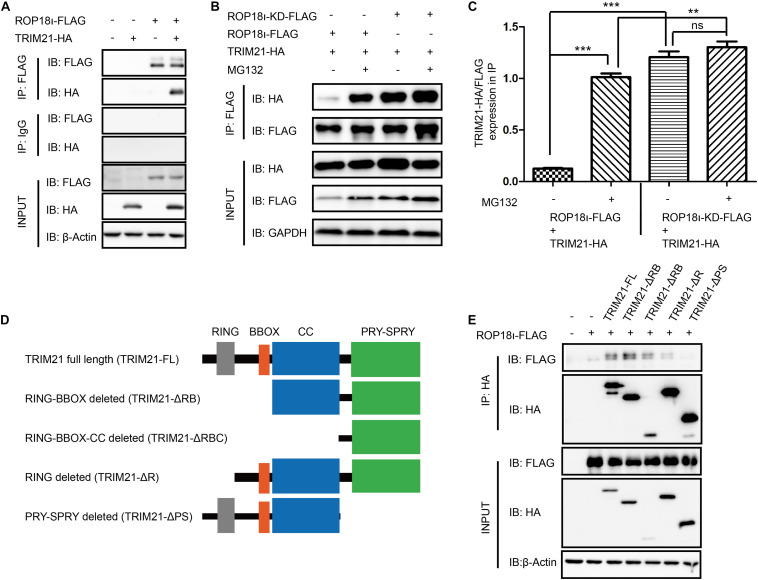
*Tg*ROP18_I_ interacted with TRIM21’s PRY-SPRY domain. **(A)** The Co-IP result indicated the interaction of *Tg*ROP18_I_ and TRIM21. **(B,C)** The Co-IP result indicated that ROP18-KD still bound with TRIM21. **(D)** Sketch map of TRIM21 WT and truncation mutants. **(E)** The Co-IP results showed the PRY-SPRY domain of TRIM21 was indispensable for *Tg*ROP18_I_-TRIM21 interaction. The experiments were repeated three times. The values were analyzed using the one-way ANOVA. Data were expressed as the mean ± SEM (**p < 0.01; ***p < 0.001; IP, immunoprecipitation).

Our co-immunoprecipitation (Co-IP) assay identified the interaction of *Tg*ROP18_I_ with human TRIM21 (Figure 1A). Meanwhile, we found that the kinase dead mutant ROP18-KD still interacted with TRIM21 (Figure 1B). Furthermore, as a potent proteasome inhibitor, MG132 treatment promoted the interaction of *Tg*ROP18_I_-TRIM21 (Figures 1B,C). As we know, TRIM21 contains four domains: RING, B box, CC (coiled-coil domain), and PRY-SPRY. We therefore further identified the domains of TRIM21 required for *Tg*ROP18_I_ interaction. We successfully constructed the truncated mutants of TRIM21 as shown in the schematic diagram (Figure 1D) using the primers shown in Supplementary Table 3. The Co-IP results showed that PRY-SPRY domain deletion resulted in *Tg*ROP18_I_ loss (Figure 1E), indicating that the PRY-SPRY domain of TRIM21 is responsible for the interaction with *Tg*ROP18_I_. Together, these results suggested that TRIM21 interacted with *Tg*ROP18_I_ *via* its PRY-SPRY domain.

### *Tg*ROP18_I_ Promoted the Phosphorylation of TRIM21

**FIGURE 2:**
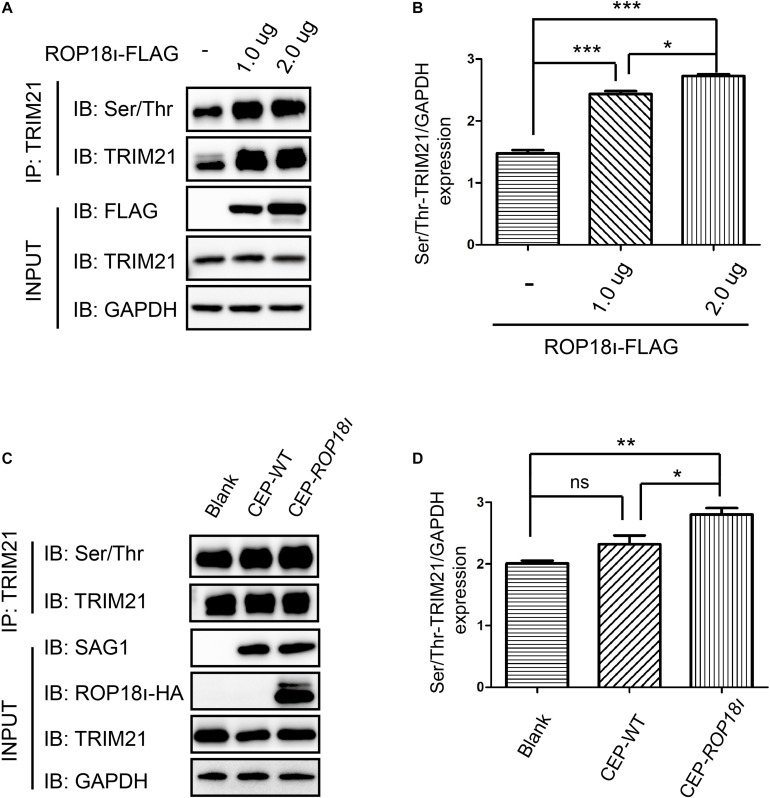
*Tg*ROP18_I_ promoted the phosphorylation of TRIM21. **(A,B)** HEK293T cells were transfected with different amount of pcDNA3.1-ROP18_I_-FLAG as indicated, and the cell lysates were subjected to TRIM21 IP and Western blotting. The more plasmids were transfected to HEK293T cells, the higher levels of phosphorylated TRIM21 were observed, and the differences between groups were significant. **(C,D)** HFF cells were infected with CEP or CEP-*rop18*_I_, and then the total protein was extracted and subjected to TRIM21 IP and Western blotting. The results showed that more phosphorylated TRIM21 was detected in the CEP-*rop18*_I_ infected cells than which detected in the CEP infected cells and uninfected cells, while the phosphorylation levels of TRIM21 were not significantly different between the CEP infected and uninfected cells. The experiments were repeated three times. The values were analyzed using the one-way ANOVA. Data were expressed as the mean ± SEM (*p < 0.05; **p < 0.01; ***p < 0.001).

Our immunoprecipitation (IP) result indicated that *Tg*ROP18_I_ overexpression in the HEK293T cells elevated the phosphorylation level of TRIM21; the more pcDNA3.1-ROP18_I_-FLAG was transfected, the more phosphorylated TRIM21 was precipitated, and the differences between groups were significant (Figures 2A,B). A consistent result was observed in CEP and CEP-*rop18*_I_ infection to HFF cells. The phosphorylation level of TRIM21 in CEP infected cells was not significantly different from that in the uninfected cells (Figures 2C,D). However, the TRIM21 phosphorylation level in CEP-*rop18*_I_ infected HFF cells was significantly higher than that in the uninfected cells and the CEP infected cells (Figures 2C,D).

### *Tg*ROP18_I_ Promoted Degradation of TRIM21 Through Lysosomal Pathway

**FIGURE 3:**
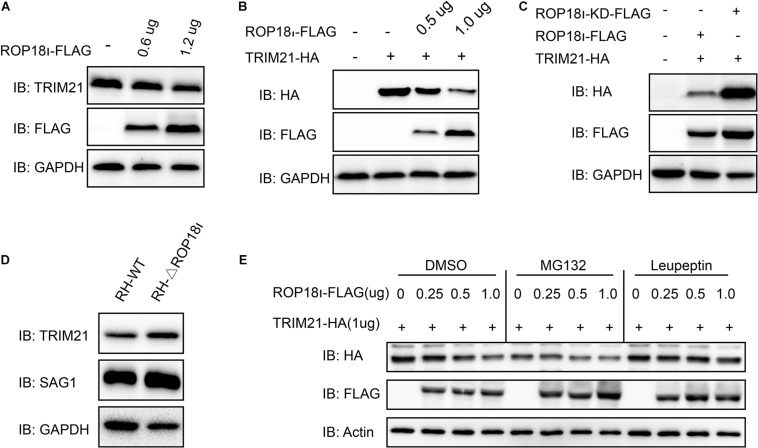
*Tg*ROP18_I_ promoted TRIM21 degradation through lysosomal pathway. **(A)** HEK293T cells were transfected with the increased amounts of pcDNA3.1-ROP18_I_-FLAG as indicated. **(B)** HEK293T cells were co-transfected with a stable amount of pcDNA3.1-TRIM21-HA and the increased amounts of pcDNA3.1-ROP18_I_-FLAG as indicated. The endogenous or overexpressed TRIM21 level was decreased with the increased ROP18 level. **(C)** HEK293T cells were co-transfected with pcDNA3.1-TRIM21-HA and pcDNA3.1-ROP18_I_-FLAG or pcDNA3.1-ROP18_I_-KD-FLAG as indicated. The results of Western blotting detection with the cell lysates indicated that much more TRIM21 was detected in the ROP18-KD overexpression group than in the ROP18 overexpression group. **(D)** Lysates of HFFs infected with RH or RH-△*rop18* was detected by Western blotting, and more TRIM21 was detected in the RH-△*rop18* infection group than in the RH infection group. **(E)** HEK293T cells were co-transfected with 1mg of pcDNA3.1-TRIM21-HA and increased amounts of pcDNA3.1-ROP18_I_-FLAG. The cells were treated with MG132 or Leupeptin, or left untreated. Cell lysates were subjected to Western blotting, and the results showed that TRIM21’s level was decreased with the increased amount of *Tg*ROP18_I_ in the cells treated with MG132 or DMSO. However, TRIM21’s level was kept stable in the Leupeptin treated group. All the experiments were repeated three times. IB, immunoblot.

We further investigated the result of TRIM21-*Tg*ROP18_I_ interaction. We found that the endogenous TRIM21 protein level was decreased with the increased *Tg*ROP18_I_ level (Figure 3A). Co-transfection of a stable amount of pcDNA3.1-TRIM21-HA with the increased amounts of pcDNA3.1-ROP18_I_-FLAG in HEK293T cells showed that, the over-expressed TRIM21 was decreased by *Tg*ROP18_I_ in a dose-dependent manner (Figure 3B). Furthermore, compared to ROP18 overexpression, the ROP18-KD overexpression resulted in less degradation of TRIM21 (Figure 3C). This result indicated that the kinase activity of *Tg*ROP18_I_ was required for *Tg*ROP18_I_-mediated TRIM21 degradation. This phenomenon was further verified by the parasitic infection experiment. HFFs were infected with RH and RH-△*rop18* strains. Comparing to RH-△*rop18* infection, RH infection led to a significant degradation of TRIM21 (Figure 3D). To identify the pathway that involved in TRIM21 degradation, HEK293T cells were co-transfected with a stable amount of pcDNA3.1-TRIM21-HA and increased amounts of pcDNA3.1-ROP18_I_-FLAG as indicated in Figure 3E. Before cell harvest, the cells were treated with MG132 or the lysosome inhibitor Leupeptin. From the results, we found that MG132 treatment had no effect on the TRIM21 degradation mediated by *Tg*ROP18_I_; however, Leupeptin treatment resulted in a stable TRIM21 protein level regardless of *Tg*ROP18_I_ existence or not (Figure 3E). In conclusion, our experiments demonstrated that *Tg*ROP18_I_ targeted human TRIM21 and resulted in TRIM21 degradation through lysosomal pathway, which was dependent on its kinase activity.

### *Toxoplasma gondii* Infection Elevated TRIM21 Transcription and Translation Level

**FIGURE 4:**
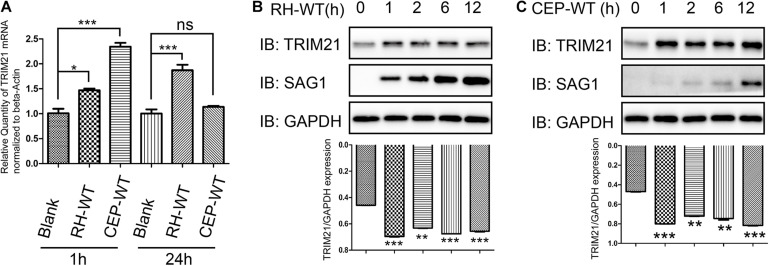
TRIM21 transcription and translation levels were upregulated following *T. gondii* infection. HFF cells infected with RH or CEP were harvested for detection of TRIM21 transcription and translation level. **(A)** Comparison of the TRIM21 transcription levels between the indicated groups with qRT-PCR. The CEP infection resulted in the highest TRIM21 transcription level, followed by RH infection, and uninfection at 1 h post infection, the differences between groups were significant. At 24 h post infection, the RH infection resulted in a significant higher TRIM21 transcription level than in CEP infection or uninfection groups between which no significant difference was observed in TRIM21 transcription level. **(B,C)** Comparison of the translation level of TRIM21 in the HFF cells infected with RH or CEP for the indicated time with Western blot (up panels). The densitometrical analysis for the intensity of TRIM21 bands normalized to its corresponding GAPDH intensity showed that, both CEP and RH infection resulted in significant higher transcription levels of TRIM21 than in uninfected cells (down panels). All the experiments were repeated four times. The values were analyzed using the one-way ANOVA. Data were expressed as the mean ± SEM (*p < 0.05; **p < 0.01; ***p < 0.001).

To investigate whether the IFN-γ inducible TRIM21 expression is regulated by *T. gondii* infection, we infected HFFs with RH or CEP strains, and evaluated the relative transcription and translation level of TRIM21. The qRT-PCR result indicated that, compared to the normal HFFs, *T. gondii* RH and CEP infection led to significantly upregulated transcription of TRIM21 at 1 h post-infection; however, with the infection going on, only *T. gondii* RH infection kept the significantly upregulated transcription of TRIM21 at 24 h post-infection, CEP infection led to a similar transcription level as in the uninfected cells (Figure 4A). Similarly, compared with the uninfected cells, a significantly higher TRIM21 protein level was detected in the HFFs infected with either RH or CEP, at 1–12 h post infection (Figures 4B,C). These results demonstrated that transcription and translation level of TRIM21 were upregulated during *T. gondii* infection in HFFs.

### TRIM21 Was Involved in the IFN-γ Induced Inhibition of CEP (but Not RH) Proliferation in HFFs

**FIGURE 5:**
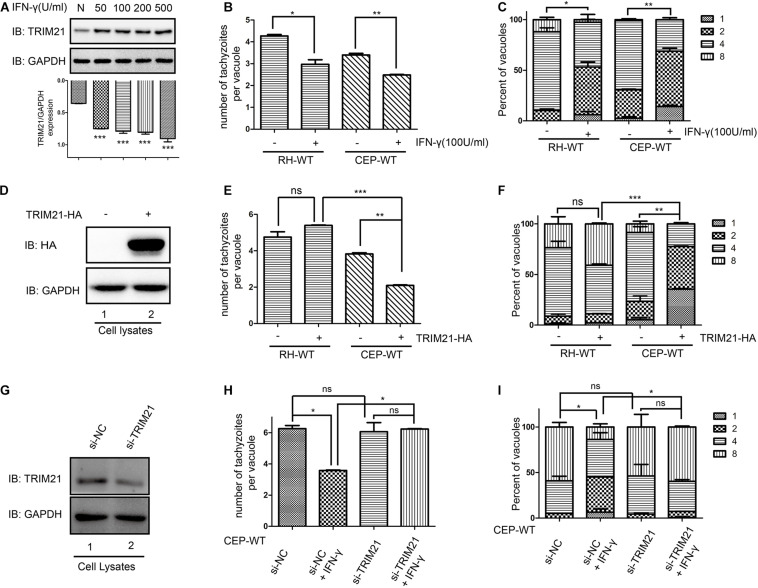
TRIM21 is involved in the IFN-γ induced inhibition of CEP proliferation (but not RH proliferation) in HFFs. The proliferation of RH and CEP tachyzoies in the HFFs was evaluated after stimulation with IFN-γ for 24 h **(A–C)**, or transfection with pcDNA3.1-TRIM21-HA for 24 h **(E–F)**; and proliferation of CEP-WT was evaluated after transfection with si-TRIM21 for 48 h in HFFs followed by IFN-γ treatment for 24 h **(H–I)**. TRIM21 protein levels were measured by Western blotting **(A,D,G)**. The average number of tachyzoites in 100 parasitophorous vacuoles (PVs) was counted **(B,E,H)**, and the percentage of the PVs containing 1, 2, 4, or 8 parasites was determined by immune fluorescence assay **(C,F,I)**. **(A)** The TRIM21 protein level was significantly up-regulated under IFN-γ stimulation in a dose-dependent manner, with the response concentration ranged from 50 to 500 U/ml. **(B,C)** The proliferation of the RH and CEP tachyzoites in HFFs was significantly inhibited after IFN-γ stimulation. **(D)** The overexpression of TRIM21 was detected. **(E,F)** The overexpression of TRIM21 significantly inhibited the CEP proliferation, but not affected RH proliferation. **(G)** The si-TRIM21 transfection significantly inhibited the TRIM21 translation. **(H,I)** The IFN-γ induced inhibition of CEP proliferation was relieved by TRIM21 knockdown. All the experiments were repeated three times. The values were analyzed using the one-way ANOVA and two-way ANOVA. Data were expressed as the mean ± SEM (*p < 0.05; **p < 0.01; ***p < 0.001).

TRIM21 protein level was upregulated after stimulation with IFN-γ regardless of the concentrations (Figure 5A). Calculating for the number of tachyzoites per vacuole (Figure 5B) and the percentages of the vacuoles containing 1, 2, 4, and 8 tachyzoites (Figure 5C), the results showed the proliferation of both RH and CEP strains was significantly inhibited following IFN-γ treatment (Figures 5B,C). We therefore wanted to know what the role of TRIM21 on *T. gondii* proliferation. HFF cells were transfected with pcDNA3.1-TRIM21-HA, and the overexpression of TRIM21 was detected by Western blot (Figure 5D); the proliferation of the parasites was determined by IFA. We found that TRIM21 overexpression resulted in the inhibition of CEP replication, but did not affect RH replication (Figures 5E,F). We next wondered if TRIM21 loss affected CEP replication, we examined the CEP replication in the HFFs with TRIM21 knockdown. We found that si-TRIM21 transfection resulted in a significantly reduced TRIM21 level compared to that in the control group (Figure 5G and Supplementary Figure 1A). Though the replication of CEP was significantly inhibited in the IFN-γ treated HFFs, but it was not affected in the TRIM21 knockdown groups regardless of IFN-γ treatment or not (Figures 5H,I and Supplementary Figures 1B,C).

### TRIM21 Knockdown Relieved the IFN-γ-Induced Ubiquitin Labeling on CEP Parasitophorous Vacuole Membrane in HFFs

**FIGURE 6:**
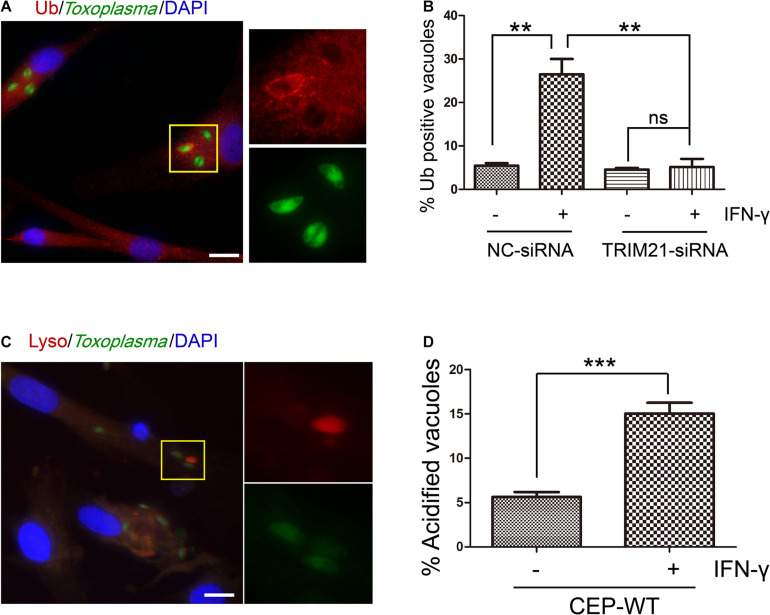
TRIM21 knockdown relieves the IFN-γ-induced ubiquitin labeling on CEP parasitophorous vacuole membrane (PVM) in HFFs. **(A,B)** HFFs were transfected with negative control siRNA (si-NC) or siRNA specific against TRIM21 (si-TRIM21), and stimulated with IFN-γ or not as indicated. After *T. gondii* infection, HFFs were subjected to immunofluorescence assay (IFA). IFN-γ induced ubiquitin labeling on the CEP PVM, but this labeling was relieved by TRIM21 knockdown regardless of IFN-γ simulation or not. **(C,D)** HFFs were stimulated with IFN-γ or not, and infected with CEP. The cells were then treated with LysoTracker^®^ (acidic dye) and subjected to immunofluorescence assay (IFA). The result showed us that IFN-γ induced acidification of CEP. On the left, a representative fluorescent image is shown for the *T. gondii* CEP strain expressing GFP. The yellow box inside each representative image is shown as magnified pictures nearby **(A,C)**. The percentage of vacuoles stained red with ubiquitin labeling or LysoTracker^®^ was shown in the right bar diagram **(B,D)**. Scale bar is 10 μm. The experiments were repeated three times. The values were analyzed using the one-way ANOVA or two-tailed unpaired Student *t* test. Data were expressed as the mean ± SEM (**p < 0.01; ***p < 0.001).

To determine whether TRIM21 was involved in IFN-γ-induced ubiquitin labeling on CEP PVM, we conducted an IFA with anti-FK-2 antibody. We found that IFN-γ induced ubiquitin labeling on the CEP PVM, but this labeling was relieved by TRIM21 knockdown regardless of IFN-γ simulation or not (Figures 6A,B). Furthermore, we investigated the outcome of the CEP tachyzoites in the PVs labeled by ubiquitin with LysoTracker^®^ staining, finding that IFN-γ induced acidification of CEP’s PV (Figures 6C,D). These results indicated that TRIM21 knockdown in HFFs relieved the ubiquitin labeling on CEP’s PV induced by IFN-γ, which would result in PV acidification to kill the parasite in it.

### *Tg*ROP18_I_ Relieved the Inhibition of *Toxoplasma gondii* Proliferation Caused by TRIM21

As we had identified that IFN-γ treatment resulted in the inhibition of RH and CEP replication, which also induced TRIM21 expression in HFFs (Figure 5). However, TRIM21 overexpression resulted in the inhibition of CEP replication, but did not affect RH replication (Figure 5); furthermore, TRIM21 knockdown relieved the IFN-γ induced inhibition of CEP (but not RH) proliferation (Figure 5). We therefore deduced that TRIM21 only functioned in CEP infection, but not in RH infection. As CEP strain does not express ROP18 as RH strain does, we speculated that the “loss of function” for TRIM21 during RH infection might be resulted by *Tg*ROP18_I_ which degraded TRIM21 through lysosomal pathway (Figure 3).

**FIGURE 7:**
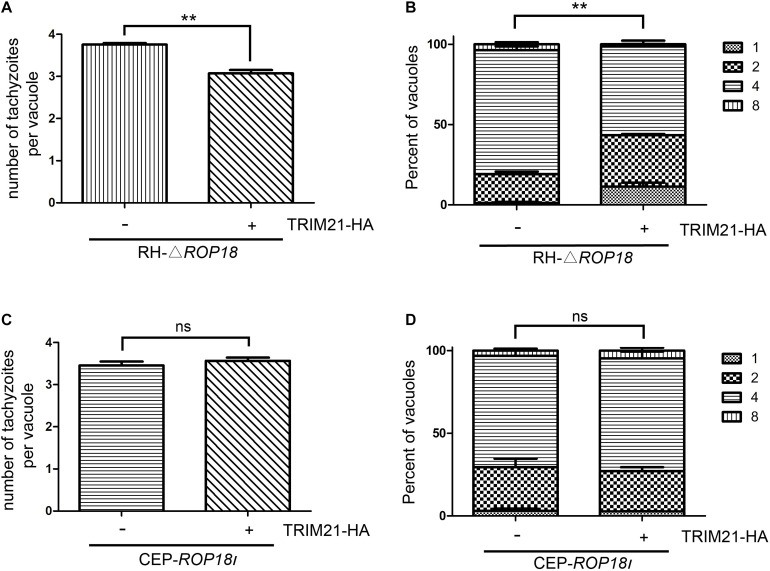
*Tg*ROP18_I_ relieved TRIM21 mediated inhibition of *T. gondii* proliferation regardless of strain types. **(A–D)** HFFs were transfected with pcDNA3.1-TRIM21-HA or pcDNA3.1(+) for control, and infected with RH-△*rop18* **(A,B)** or CEP-*rop18*_I_ **(C,D)** parasites as indicated. Parasitic proliferation was measured at 18 h (RH-△*rop18*) or 24 h (CEP-*rop18*_I_) post-infection. The average number of tachyzoites in 100 vacuoles **(A,C)** or the number of vacuoles containing 1, 2, 4, or 8 parasites **(B,D)** was determined by fluorescence microscopy. The results indicated that TRIM21 overexpression inhibited the RH-△*rop18* multiplication, but had no significant effect on CEP-*rop18*_I_ multiplication. The experiments were repeated three times. The values were analyzed using the two-tailed unpaired Student *t* test and two-way ANOVA. Data were expressed as the mean ± SEM (**p < 0.01).

To assess whether upregulated TRIM21 can be reversed by *Tg*ROP18_I_ during RH infection, we conducted a proliferation assay with RH-△*rop18* and CEP-*rop18*_I_ strains. The result showed that TRIM21 overexpression resulted in the inhibition of RH-△*rop18* proliferation (Figures 7A,B), but had no effect on CEP-*rop18*_I_ proliferation (Figures 7C,D). These results confirmed that TRIM21 medicated inhibition of *T. gondii* proliferation was relieved by *Tg*ROP18_I_, and was irrelevant to *T. gondii* strain types.

### TRIM21 Medicated NF-κB Activation

**FIGURE 8:**
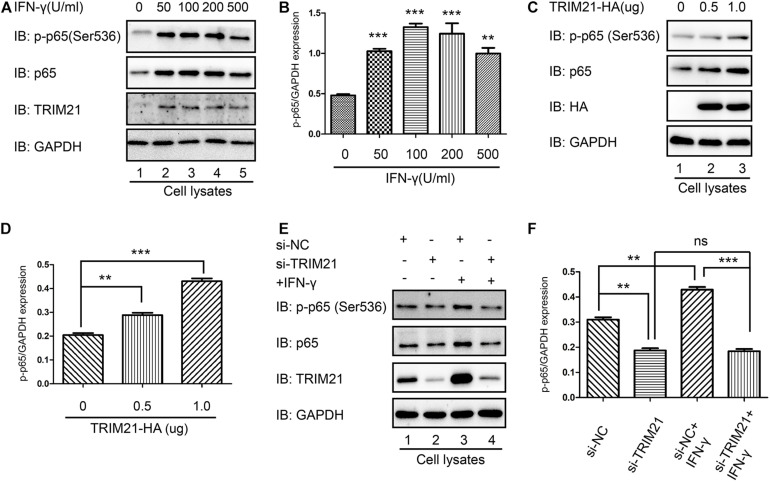
TRIM21 mediated NF-κB activation. **(A,B)** In the HFF cells stimulated with the indicated concentrations of IFN-γ for 24 h, the p-p65 (S536) level was significantly higher than that in the untreated group. **(C,D)** Western-blot detection of p-p65 (S536) level showed that TRIM21 overexpression significantly elevated p-p65 (S536) phosphorylation in dose-dependent manner. **(E,F)** In the siRNA-TRIM21 transfected groups, TRIM21 expression was suppressed, but no significant difference was found in the p-p65 (S536) levels between the TRIM21 knockdown group with or without IFN-γ induction. The experiments were repeated three times. The values were analyzed using the one-way ANOVA and the data were expressed as the mean ± SEM (**p < 0.01; ***p < 0.001).

As p65 phosphorylation represents the activation of NF-κB, both p-p65 (Ser536) and TRIM21 levels were upregulated by IFN-γ treatment in HFF cells, compared to which in the untreated cells (Figures 8A,B). To further reveal the relation between TRIM21 and NF-κB, we overexpressed TRIM21 in HFFs to detect p65 (S536) phosphorylation. The result showed us that the p-p65 (S536) level was significantly upregulated (representing significant NF-κB activation) by TRIM21 overexpression in dose-dependent manner (Figures 8C,D). After that, HFFs were transfected with si-TRIM21, and the TRIM21 knockdown was verified in si-TRIM21 transfected cells (Figure 8E). The result indicated that the phosphorylation of p65 (Ser536) was downregulated by TRIM21 knockdown (Figures 8E,F). We therefore concluded that TRIM21 functioned in NF-κB activation.

### TRIM21 Blocked the Interaction of p65 and IκB-α

**FIGURE 9:**
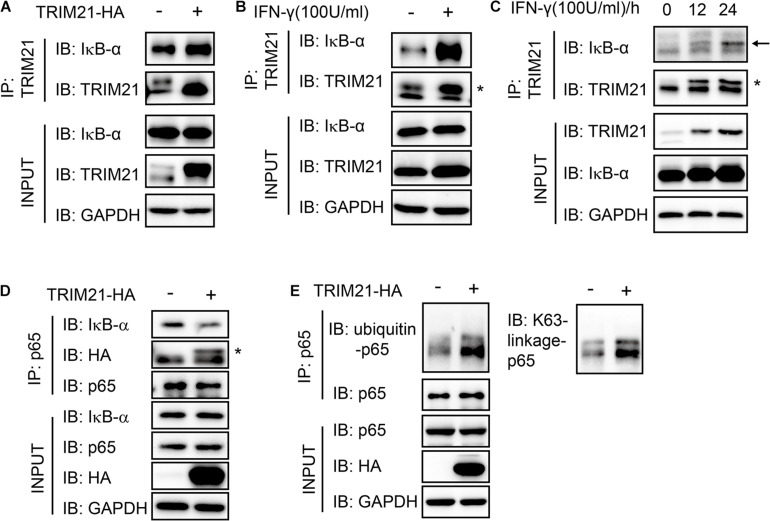
The interaction of p65 and IκB-α was suppressed with TRIM21 overexpression. Interaction of TRIM21 and IκB-α in the total cell lysates of HEK293T cells were analyzed by IP and Western-blot after either treatment with IFN-γ or transfection with pcDNA3.1-TRIM21-HA for the indicated time. **(A–C)** The IP with the anti-TRIM21 antibody identified the interaction of IκB-α with the overexpressed and the endogenous TRIM21. TRIM21 overexpression promoted TRIM21-IκB-α interaction **(A)**. IFN-γ induction elevated TRIM21 production and promoted TRIM21-IκB-α interaction **(B)**. The TRIM21 production induced by IFN-γ increased with the prolonged treating time and promoted TRIM21-IκB-α interaction **(C)**. **(D,E)** The IP with the anti-p65 antibody identified the complex of IκB-α-TRIM21-p65 **(D)**, and the interaction of p65-ubiquitin **(E)**. The experiments were repeated three times. The TRIM21 band is indicated with “*”, and the IκB-α band is indicated with a black arrow.

As the interaction of p65 and IκB-α was essential for NF-κB activation, we next sought to find out whether their interaction regulated by TRIM21. We firstly performed immunoprecipitation with anti-TRIM21 antibody. The result showed that TRIM21 interacted with IκB-α and TRIM21 overexpression promoted the interaction of TRIM21 and IκB-α, this phenomenon was also confirmed with IFN-γ stimulation (Figures 9A,B). Furthermore, their interaction was promoted with the prolonged IFN-γ treating time (Figure 9C). Moreover, the complex of IκB-α-TRIM21-p65 was detected after immunoprecipitation with anti-p65 antibody, and TRIM21 overexpression resulted in inhibition of IκB-α and p65 interaction in the HEK293T cells, for less IκB-α was detected in the over-expression cells than in the normal cells (Figure 9D). Since TRIM21 is an E3 ubiquitin ligase, we next examined the p65 ubiquitination level in the TRIM21 overexpressed HEK293T cells. An increased ubiquitinated p65 level was observed in the TRIM21 overexpression cells (Figure 9E). Moreover, the result showed us that the p65-ubiquitin was mainly of K63 linkage type (Figure 9E). Together, these results suggested that TRIM21 interacted with p65 and IκB-α, so as to inhibit the interaction of p65 and IκB-α and promote p65-K63 ubiquitination. As a result, NF-κB activation was inhibited.

## Discussion

TRIM21 was originally identified as an autoantigen in autoimmune diseases, including rheumatoid arthritis, Systemic lupus erythematosus, and Sjogren’s syndrome (Schulte-Pelkum et al., 2009). TRIM21 acts as an important factor involved in the innate immune responses against microbial infection, including bacterial, viral, and parasite (Hos et al., 2020; Mu et al., 2020; Xie et al., 2020), however, little about TRIM21 is known for parasites infection. In this study, we reported that *T. gondii* RH and CEP infection upregulated the expression of TRIM21, thus restricted parasite replication through NF-κB activation. Moreover, TRIM21 suppressed the p65-IκB-α interaction to activate NF-κB pathway through binding with IκB-α.

Interferon gamma (IFN-γ) is the major cytokine responsible for controlling *T. gondii* infection (Suzuki et al., 1988; Yap and Sher, 1999; MacMicking, 2012). Previous studies reported that TRIM21 can be upregulated by pathogens and suppress pathogens by interacting with the unique host factor, causing the ubiquitination and degradation of the pathogens through the proteasome pathway (Vaysburd et al., 2013; Rhodes and Isenberg, 2017). As an interferon stimulating gene (Rhodes et al., 2002), TRIM21 level was upregulated after stimulation with IFN-γ or *T. gondii* infection. We found that *T. gondii* proliferation was inhibited by IFN-γ stimulation, which is consistent with the other reports (Suzuki et al., 1988; Yap and Sher, 1999).

Our research revealed one of the mechanisms for the different outcomes when human cells were infected by type I and type III *T. gondii*. The virulence factor discharged by intruder has become one of the most effective ways for immune escape (Ashida et al., 2010; Sanada et al., 2012; Zhou and Zhu, 2015). During *S. typhimurium* infection, SPI-1-encoded effector protein SopA discharged by bacteria promoted TRIM65 and TRIM56 ubiquitination and degradation (Fiskin et al., 2017). *Tg*ROP18_I_, as the key virulence factor during *T. gondii* infection (El Hajj et al., 2007), is a Ser/Thr kinase of ROP2 subfamily, which interacted and phosphorylated host immune factor, including p65 (Du et al., 2014), p53, p38, UBE2N and Smad1 (Yang et al., 2017). In this study, we found the interaction of *Tg*ROP18_I_ with human TRIM21 on the PRY-SPRY domain of TRIM21, and it promoted TRIM21 phosphorylation, and resulted in TRIM21 degradation through lysosomal way. We identified that IFN-γ treatment resulted in the inhibition of RH and CEP replication, and induced TRIM21 expression in HFFs. However, TRIM21 overexpression resulted in the inhibition of CEP replication, but did not affect RH replication; on the other hand, TRIM21 knockdown relieved the IFN-γ induced inhibition of CEP (but not RH) proliferation. Therefore, we deduced that TRIM21 only functioned in CEP infection, but not in RH infection. *Tg*ROP18_I_ may be the reason for the different responses of host cells mediated by TRIM21 during RH and CEP infection.

Furthermore, our proliferation assay conducted with RH-△*rop18* and CEP-*rop18*_I_ strains showed that TRIM21 overexpression resulted in the inhibition of RH-△*rop18* proliferation, but had no effect on CEP-*rop18*_I_ proliferation. A recent study reported that deletion of TRIM21 gene promoted *T. gondii* replication (Foltz et al., 2017), while the effect of TRIM21 overexpression on *T. gondii* infection needed further investigations. Therefore, we concluded that TRIM21 medicated inhibition of *T. gondii* proliferation was relieved by *Tg*ROP18_I_, and was irrelevant to strain types.

TRIM family members have been regarded as key factors of innate immunity (Giraldo et al., 2020; Koepke et al., 2020). Accumulating studies have been reported that some members of the TRIM family positively regulated the NF-κB pathway (Kawai and Akira, 2011; Li et al., 2011; Hu et al., 2014). For instance, a recent study suggested that TRIM13 interfered TNF receptor associated factor 6 (TRAF6) to upregulate NF-κB activity (Huang and Baek, 2017). TRIM21 has been shown to stimulate NF-κB pathway activation (Yang et al., 2020). Moreover, IκB-α binds p65 subunit, which inhibits NF-κB activation and the IκB proteins are degraded, which promotes p65 entering the nucleus to activate NF-κB (Scheidereit, 2006). In consistence with these reports, we found that TRIM21 overexpression suppressed the p65-IκB-α interaction to promote the activation of NF-κB, which resulted in the inhibition of CEP proliferation. On the other hand, a suppressed NF-κB activation was observed in TRIM21 knockdown HFFs after CEP infection, but had no effects on CEP proliferation.

We also found that the IFN-γ-induced ubiquitin labeling of PVM was tightly controlled by TRIM21. Ubiquitin contains seven lysine residues that can form polyubiquitin chains (Komander and Rape, 2012). The formation of ubiquitin chains at different lysine residues leads to distinct outcomes (Zhao and Ulrich, 2010). Lysine 63 (K63) polyubiquitination to substrates is important for activation of signal transduction (Zhao and Ulrich, 2010; Komander and Rape, 2012). In our study, we identified that TRIM21 interacted with p65 and increased the p65 K63-ubiquitination level to promote NF-κB activation, which may be a possible mechanism for TRIM21 enhancing NF-κB activation to inhibit the proliferation of *T. gondii*. Ubiquitin recognition of intracellular bacteria and virus has become the hallmark event to mediate cellular immune response for restriction of the intruders (Heaton et al., 2016; Kuo et al., 2018; Wang et al., 2018). The coating of intracellular pathogens with ubiquitin is considered as a conserved defense mechanism from fruit flies to humans (Boyle and Randow, 2013; Manzanillo et al., 2013). Ubiquitin recognition of the substrates depends on the specificity of E3 ubiquitin ligases, and it’s important to screen the specific E3 ubiquitin ligases responsible for the interaction of host and pathogens. For instance, *Salmonella* was directly recognized by an E3 ubiquitin ligase, leucine rich repeat and sterile alpha motif containing 1 (LRSAM1), which led to the ubiquitination and autophagy of the bacterium (Huett et al., 2012). During *T. gondii* infection, K63-ubiquitin recognition of PVM conferred it to acidic destruction and proliferation obstacle in human umbilical vein endothelial cells (HUVEC) (Clough et al., 2016). We also found that TRIM21 knockdown relieved the ubiquitin labeling on PVM in IFN-γ primed HFFs, which indicated that TRIM21, as an E3 ubiquitin ligase, contributed to the IFN-γ-induced ubiquitin immune response against *T. gondii* infection.

**FIGURE 10:**
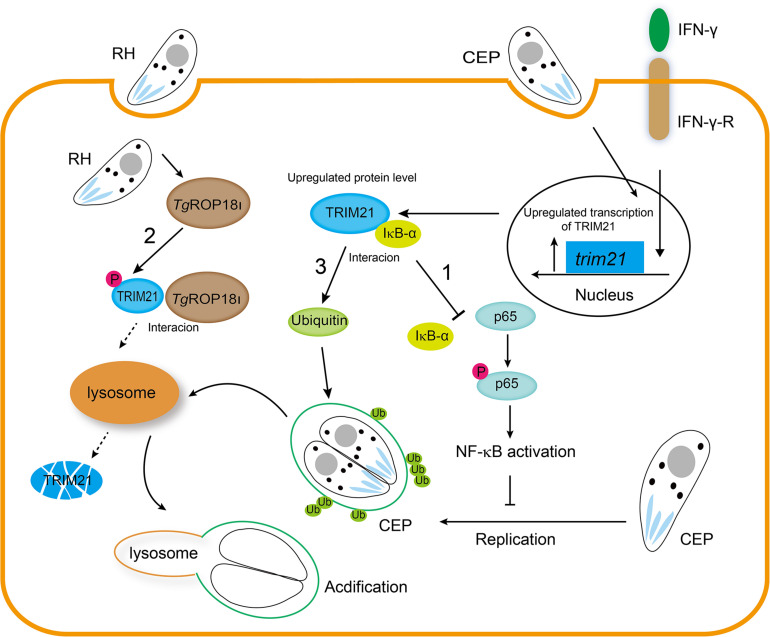
*Tg*ROP18_I_ targets host TRIM21 for immune escape. 1. Host IFN-γ-induced factor TRIM21 restricted *T. gondii* replication through NF-κB activation and TRIM21 overexpression suppressed the p65-IκB-α interaction to activate NF-κB pathway. 2. *Tg*ROP18_I_ which was discharged by *T. gondii*, interacted with the PRY-SPRY domain of human TRIM21, promoted TRIM21 phosphorylation, and induced TRIM21 degradation *via* lysosomal pathway. 3. IFN-γ induced ubiquitin labeling on the CEP PVM which resulted in PV acidification and death of parasites, but this labeling was relieved by TRIM21 knockdown regardless of IFN-γ simulation or not.

In conclusion, our findings are highlighted as follows (Figure 10). 1. Host IFN-γ-induced factor TRIM21 restricted *T. gondii* replication through NF-κB activation and TRIM21 overexpression suppressed the p65-IκB-α interaction to activate NF-κB pathway. 2. *Tg*ROP18_I_ which was discharged by *T. gondii*, interacted with the PRY-SPRY domain of human TRIM21, promoted TRIM21 phosphorylation, and induced TRIM21 degradation *via* lysosomal pathway. 3. IFN-γ induced ubiquitin labeling on the CEP PVM which resulted in PV acidification and death of parasites, but this labeling was relieved by TRIM21 knockdown regardless of IFN-γ simulation or not.

## Data Availability Statement

The original contributions presented in the study are included in the article/Supplementary Material, further inquiries can be directed to the corresponding author/s.

## Author Contributions

All authors listed have made a substantial, direct and intellectual contribution to the work, and approved it for publication.

## Conflict of Interest

The authors declare that the research was conducted in the absence of any commercial or financial relationships that could be construed as a potential conflict of interest.

## Supplementary Material

Supplementary Figure 1 The proliferation of RH was measured after TRIM21 knockdown for 48 h following IFN-γ stimulation for 24 h. **(A)** TRIM21 protein levels were measured by Western blotting. **(B,C)** The average number of tachyzoites in 100 parasitophorous vacuoles (PVs) was counted **(B)**, and the percentage of the PVs containing 1, 2, 4, or 8 parasites was determined by fluorescence microscope **(C)**. TRIM21 knockdown following IFN-γ had no significant effect on the proliferation of RH tachyzoites in HFF cells. The experiments were repeated three times. The values were analyzed using the one-way ANOVA. Data were expressed as the mean ± SEM (^∗∗^*p* < 0.01).

Supplementary Table 1 The gene names and sequences used for q-RT-PCR analysis.

Supplementary Table 2 The siRNA sequences used for TRIM21 knockdown.

Supplementary Table 3 The primers used for construction of the full length and truncation mutants of TRIM21 with HA-tag.
