# Supplementary figures and images for: Toxoplasma gondii Type-I ROP18 Targeting Human E3 Ligase TRIM21 for Immune Escape
Source: Front Cell Dev Biol. 2021 May 26;9:685913. doi: 10.3389/fcell.2021.685913 (PMC8187923; doi:10.3389/fcell.2021.685913)

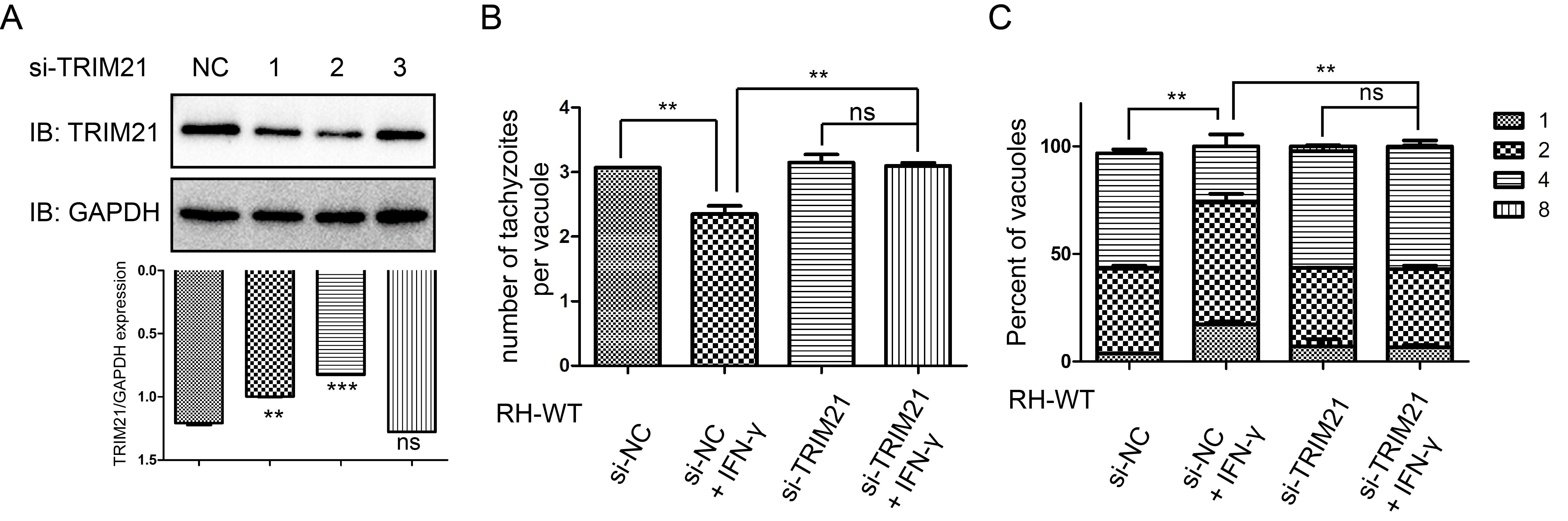

Supplement: Supplementary Figure 1 — The proliferation of RH was measured after TRIM21 knockdown for 48 h following IFN-γ stimulation for 24 h. (A) TRIM21 protein levels were measured by Western blotting. (B,C) The average number of tachyzoites in 100 parasitophorous vacuoles (PVs) was counted (B), and the percentage of the PVs containing 1, 2, 4, or 8 parasites was determined by fluorescence microscope (C). TRIM21 knockdown following IFN-γ had no significant effect on the proliferation of RH tachyzoites in HFF cells. The experiments were repeated three times. The values were analyzed using the one-way ANOVA. Data were expressed as the mean ± SEM (∗∗p < 0.01). [file Image_1.JPEG]
