# Supplementary material for: Toxoplasma gondii Type-I ROP18 Targeting Human E3 Ligase TRIM21 for Immune Escape
Source: Front Cell Dev Biol. 2021 May 26;9:685913. doi: 10.3389/fcell.2021.685913 (PMC8187923; doi:10.3389/fcell.2021.685913)
Supplement: Supplementary Table 1 — The gene names and sequences used for q-RT-PCR analysis. [file Table_1.DOCX]

Supplementary Materials and Methods

**Table S1** The gene names and sequences used for q-RT-PCR analysis.

| Gene name | Forward ( 5'→3' ) | Reverse ( 5'→3' ) |
| --- | --- | --- |
| Trim21_ 109092 | CATGCCCTATCTGCCTGGAC | TTGGGCCGGAGATTCTTGAG |
| β-Actin | GGCTACAGCTTCACCACCAC | TGCGCTCAGGAGGAGC |

**Table S2 The siRNA sequences used for TRIM21 knockdown.**

| Name | sequence (5'→3') |
| --- | --- |
| si-TRIM21-1 | TGCATCTCTCAGGTTGGGA |
| si-TRIM21-2 | GCAGCACGCTTGACAATGA |
| si-TRIM21-3 | GGACAATTTGGTTGTGGAA |

**Table S3** The primers used for construction of the full length and truncation mutants of TRIM21 with HA-tag.

| Gene name | Forward ( 5'→3' ) | Reverse ( 5'→3' ) |
| --- | --- | --- |
| TRIM21 full length (TRIM21-FL) | CCGGAATTCATGGCTTCAGCAGCACGCTT | TGCTCTAGATCAGGCATAATCGGGTACATCGTAAGGGTAATAGTCAGTGGATCCTTGTG |
| RING-BBOX deleted (TRIM21-ΔRB) | CCGGAATTCATGGAGGAGGCTGCACAGGAG | TGCTCTAGATCAGGCATAATCGGGTACATCGTAAGGGTAATAGTCAGTGGATCCTTGTG |
| RING-BBOX-CC deleted (TRIM21-ΔRBC) | CCGGAATTCATGTCCTGGAACCTGAAGGAC | TGCTCTAGATCAGGCATAATCGGGTACATCGTAAGGGTAATAGTCAGTGGATCCTTGTG |
| RING deleted (TRIM21-ΔR) | CCGGAATTCATGCTGCTCAAGAATCTCCG | TGCTCTAGATCAGGCATAATCGGGTACATCGTAAGGGTAATAGTCAGTGGATCCTTGTG |
| PRY-SPRY deleted (TRIM21-ΔPS) | CCGGAATTCATGGCTTCAGCAGCACGCTT | TGCTCTAGATCAGGCATAATCGGGTACATCGTAAGGGTAACATGTCCTCAGCATCTTC |

The underlined text indicates the HA label.
